# [Corrigendum] ND‑09 inhibits chronic myeloid leukemia K562 cell growth by regulating BCR‑ABL signaling

**DOI:** 10.3892/or.2023.8518

**Published:** 2023-03-03

**Authors:** Yan-Hong Liu, Man Zhu, Pan-Pan Lei, Xiao-Yan Pan, Wei-Na Ma

Oncol Rep 46: 136, 2021; DOI: 10.3892/or.2021.8087

Subsequently to the publication of this paper, the authors have realized that an error was made during the compilation of Fig. 2A as it appeared on p. 4. Essentially, the partial Q2-3 images of the ‘1.56 µm’ group were inadvertently copied across to the Q2-3 images of the ‘3.12 µm’ group, leading to the cell number of the Q2-3 quadrant being the same for both the 1.56 µm and the 3.12 µm groups, and also leading the total cell number of the 3.12 µm group being calculated as 106.97%, which was clearly incorrect (the total percentage should have added up to 100%).

The corrected version of Fig. 2, showing the correct data for the Q2-3 images in the ‘3.12 µm’ group, is shown on the next page. Note that this error did not significantly affect the results or the conclusions reported in this paper, and all the authors agree with the publication of this Corrigendum. The authors are grateful to the Editor of *Oncology Reports* for allowing them this opportunity to publish a corrigendum, and apologize to the readership for any inconvenience caused.

## Figures and Tables

**Figure 2.**
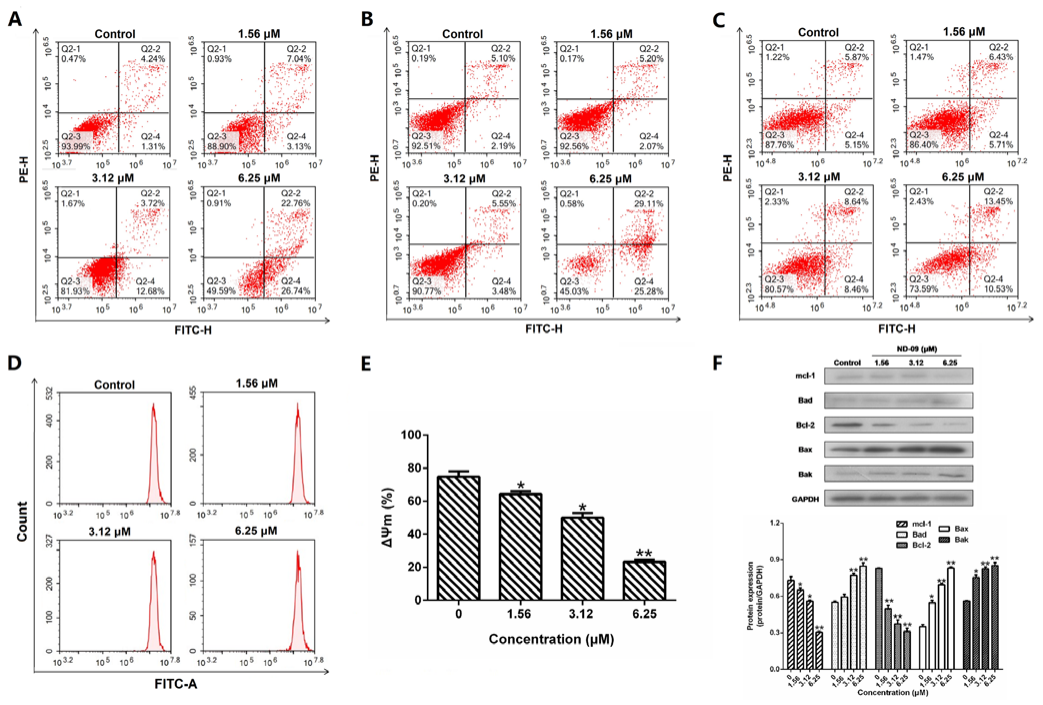
Effect of ND-09 treatment on cell apoptosis. The proportion of apoptotic cells was determined by double staining with Annexin V/FITC and PI in (A) K562, (B) JURKAT, and (C) HUT78 cells after treatment with ND-09 (0, 1.56, 3.12, and 6.25 µM). (D) Effect of ND-09 on mitochondrial membrane potential (Δψm). Δψm was assessed through flow cytometry following treatment of K562 cells with ND-09 (0, 1.56, 3.12, and 6.25 µΜ) for 48 h. (E) Quantitative analysis of flow cytometry data. (F) Effects of ND-09 on apoptosis-related protein expression in K562 cells. All results were quantified by densitometric analysis of the bands and were normalized to GAPDH (internal control). Samples were derived from the same experiment, and blots were processed in parallel. Values represent the average of three independent experiments. Data are presented as the mean ± SEM (n=3). *P<0.05, **P<0.01 compared with the untreated control group.

